# Variant Ranker: a web-tool to rank genomic data according to functional significance

**DOI:** 10.1186/s12859-017-1752-3

**Published:** 2017-07-17

**Authors:** John Alexander, Dimitris Mantzaris, Marianthi Georgitsi, Petros Drineas, Peristera Paschou

**Affiliations:** 1 Department of Molecular Biology and Genetics, Democritus University of Thrace, Panepistimioupoli, Dragana, Alexandroupolis, 68100 Greece; 2 Department of Medicine, Aristotle University of Thessaloniki, Thessaloniki, 54124 Greece; 3 Department of Computer Science, Purdue University, West Lafayette, 47907 Indiana United States; 4 Department of Biological Sciences, Purdue University, West Lafayette, 47907 Indiana United States

**Keywords:** Next-generation sequencing, Ranking, Prioritisation

## Abstract

**Background:**

The increasing volume and complexity of high-throughput genomic data make analysis and prioritization of variants difficult for researchers with limited bioinformatics skills. *Variant Ranker* allows researchers to rank identified variants and determine the most confident variants for experimental validation.

**Results:**

We describe *Variant Ranker*, a user-friendly simple web-based tool for ranking, filtering and annotation of coding and non-coding variants. *Variant Ranker* facilitates the identification of causal variants based on novelty, effect and annotation information. The algorithm implements and aggregates multiple prediction algorithm scores, conservation scores, allelic frequencies, clinical information and additional open-source annotations using accessible databases via ANNOVAR. The available information for a variant is transformed into user-specified weights, which are in turn encoded into the ranking algorithm. Through its different modules, users can (i) rank a list of variants (ii) perform genotype filtering for case-control samples (iii) filter large amounts of high-throughput data based on user custom filter requirements and apply different models of inheritance (iv) perform downstream functional enrichment analysis through network visualization. Using networks, users can identify clusters of genes that belong to multiple ontology categories (like pathways, gene ontology, disease categories) and therefore expedite scientific discoveries. We demonstrate the utility of *Variant Ranker* to identify causal genes using real and synthetic datasets. Our results indicate that *Variant Ranker* exhibits excellent performance by correctly identifying and ranking the candidate genes

**Conclusions:**

*Variant Ranker* is a freely available web server on http://paschou-lab.mbg.duth.gr/Software.html. This tool will enable users to prioritise potentially causal variants and is applicable to a wide range of sequencing data.

## Background

Identifying causal variants is critical to understanding the pathogenesis of diseases. With the advancement in high-throughput next-generation genomic technology, whole genome sequencing, exome sequencing, RNA-Seq and ChIP-Seq are now becoming standard for identifying susceptibility loci in complex and Mendelian disorders. The challenge lies in sifting through the vast amount of data these techniques generate to identify causal variants. In addition to this, researchers often face the dilemma of not knowing which is the “optimal” algorithm to use for prediction of deleteriousness (e.g.’s PolyPhen [1], SIFT [2], MutationTaster [3]) and conservation (e.g.’s PhyloP [4], SiPhy [5], GERP [6]), as there exists considerable variability in predictions from different tools. Furthermore, annotations of variant functionality tend to vary from one database to the other. There are several very useful tools for annotation of variants like SnpEff [7], SeattleSeq [8] or ANNOVAR [9] however they lack the ability to rank variants. Tools like like eXtasy [10] and SPRING [11] are limited to ranking non-synonymous variants alone. In other cases, tools like VAAST [12] and KGGSeq [13] are useful command line tools to prioritize disease-causing variants but typically the user will need some level of programming knowledge to download and execute the tools.

We have developed a web based bioinformatics tool, *Variant Ranker* to address current challenges in interpreting genomic data by providing a simple method to combine predictions and annotations of variants from various algorithms and databases respectively. The end result is a ranked list of variants to take forward for functional studies or experimental validation. Using this tool, a ranked list of prioritized variants is generated by computing a single score combining existing and available information present for a variant from several databases. *Variant Ranker* is applicable to all types of sequencing data using the *de facto*VCF [14] and ANNOVAR [9]) formats. The advantages of this tool are the ease of use, ability to score all variants (coding and non-coding) and flexibility in filtering offered to the user. Users can query results quickly through the database, thus providing easily accessible and interpretable outputs, including for those with limited bioinformatics skills. For the purpose of downstream functional enrichment analysis to discover vital biological connections from a ranked list of variants/genes, the *Network Analyser* is integrated; a network visualization tool that investigates tabular results from DAVID (database for annotation, visualization and integrated discovery, https://david.ncifcrf.gov) [15, 16] through a network approach.

## Implementation

**Fig. 1:**
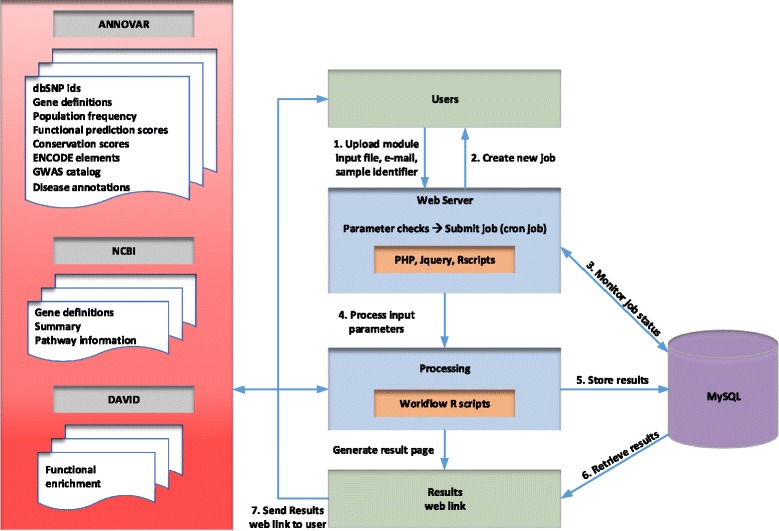
*Variant Ranker* system architecture and workflow

**Fig. 2:**
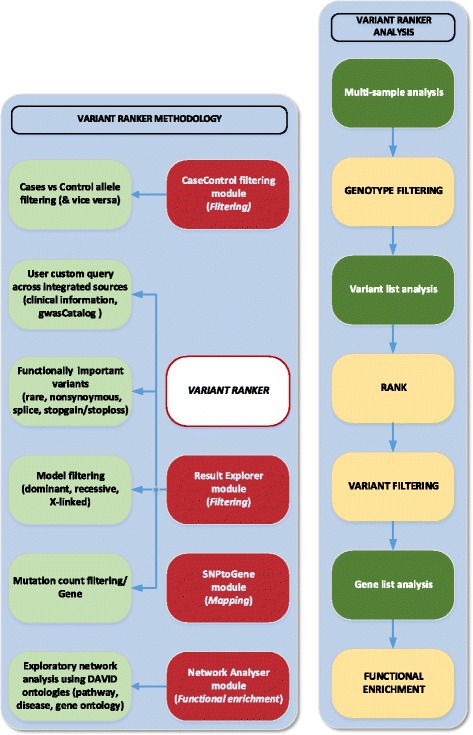
*Variant Ranker’s* functionality along with its available modules for variant/gene list analysis

The user-friendly website is constructed on an Apache web server and exploits a MySQL database using PHP, JQuery and R. Figure 1 depicts the *Variant Ranker* system architecture and workflow. Figure 2 depicts *Variant Ranker*’s functionality along with its available modules for variant/gene list analysis. We provide online tutorials with example analysis for using *Variant Ranker* and its available modules.

### Variant annotation

**Fig. 3:**
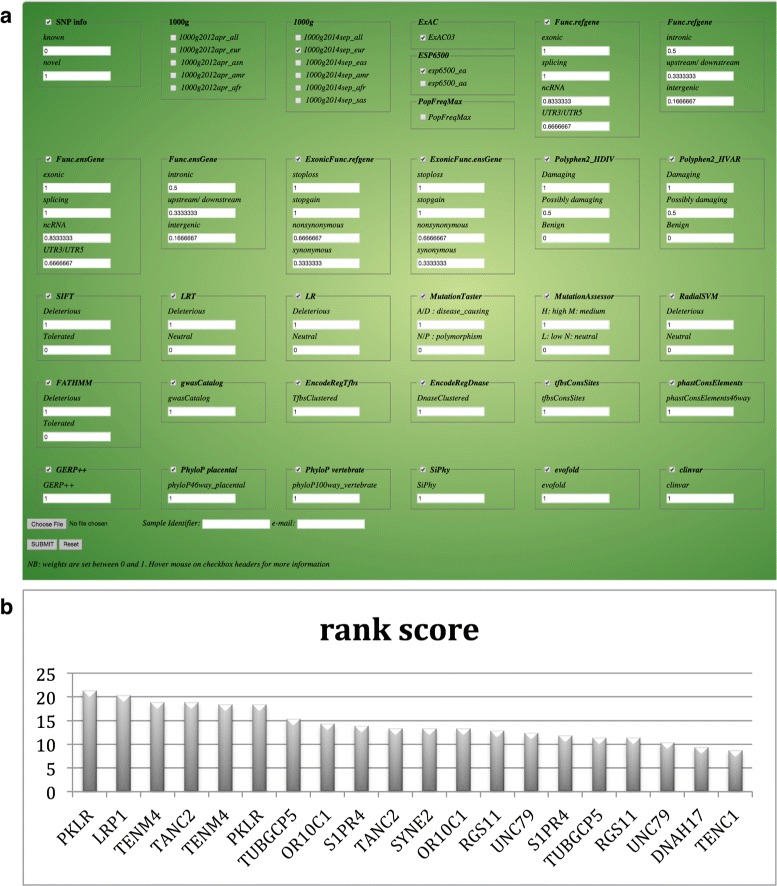
**a** *Variant Ranker* input parameter page showing default weights. These weights can be changed by the user. **b** Top 20 candidate genes from analysing an exome of an individual having idiopathic hemolytic anemia (IHA) for which *PKLR* was identified as the most likely causative gene

To facilitate the combination of various prediction algorithms and annotations, we use the annotations of variants from software ANNOVAR [9](see Fig. 3 a). Encoding annotations include: (i) Variant position and dbSNP IDs, (ii) Population frequency - rare or novel variants from 1000 Genomes Project [17], Exome Sequencing Project [18] and Exome Aggregation Consortium (ExAC) [19], (iii) Gene annotations from RefSeq [20] and ENSEMBL [21] including variant classifications like intronic/ncRNA/UTRs/exonic (nonsynonymous/stoploss/stopgain etc.), (iv) Functional prediction scores (SIFT [2], PolyPhen2 [1], LRT [22], MetaLR [23], MetaSVM [23], MutationTaster [3], MutationAssessor [24] and FATHMM [25]), (v) Conservation scores (PhyloP [4], GERP++ [6], phastCons [26], SiPhy [5]), (vi) Encoding elements from ENCODE [27], and (vii) Disease annotations (GWAS catalog [28] and clinVar [29]). Scores from CADD [30] are also included in the ranking output.

### Variant ranking algorithm

Using available annotations, all the variants are encoded by assigning weights between 0 and 1. For example, a variant is given weights following the ANNOVAR annotation precedence rule: exonic=splicing >ncRNA >UTR5/UTR3 >intron >upstream/downstream >intergenic and will have corresponding weights 1, 5/6, 4/6, 3/6, 2/6, and 1/6 respectively. Scores from conservation and prediction algorithms are converted to corresponding weights using each algorithm-scoring cut off. For example, if a variant has GERP [6] score>2 (highly conserved), it is given a corresponding weight of 1 otherwise 0. Similarly for prediction algorithm Polyphen2, weights follow 1 (damaging), 0.5 (possibly damaging) and 0 (benign) and SIFT [2], LRT [22], MetalLR [23], MetaSVM [23], MutationTaster [3], MutationAssessor [24], and FATHMM [25] follow weights 1 (deleterious) and 0 (tolerated). Binary weights (1 or 0) are applied to variants carrying ENCODE [27] elements, transcription factor binding sites or conserved sites and also if absent from dbSNP or present in the GWAS catalog [28]) or clinVAR [29] database. For population frequency databases, weights are assigned (1 – allele frequency) in order to assign more weight to rare alleles.

A higher score is thus given for a functionally important variant which is novel and predicted to be deleterious by several prediction algorithms (different algorithms tend to have different predictions). The total score for each variant is obtained by taking the sum of encoded weights per variant, and then all variants are sorted by their total score and ranked. Implementing such a score overcomes annotation discrepancies from various databases wherein a variant might be called exonic in one and intronic in the other or prediction scores may range from deleterious to tolerant from program to program. This also has the advantage of having a single score for all variants based on the available information per variant.

## Results

To demonstrate the utility of *Variant Ranker*, we applied the tool to both real exome sequencing and synthetic exome datasets. Our results indicate that *Variant Ranker* exhibits excellent performance by correctly identifying and ranking the candidate genes. For fully ranked annotation results see http://paschou-lab.mbg.duth.gr/html5up/Examples.html

### Analysis of a real exome sequencing dataset on idiopathic hemolytic anemia (MIM: 266200)

We used the exome of an individual with idiopathic hemolytic anemia (IHA) for which *PKLR* was identified as the most likely causative gene [31, 32]. 28,644 variants were ranked reporting *PKLR* as the 4th rank. On applying further filtering using the autosomal rare recessive model, the number of variants reduced to 28 with *PKLR* as the top candidate gene (out of 14 candidate genes) Fig. 3 b.

### Analysis of synthetic whole-genome sequencing dataset on Pfeiffer syndrome (MIM: 101600)

We supplemented the p.E173A mutation into a normal exome VCF file containing 33,862 variants in the *FGFR2* gene associated with Pfeiffer syndrome (MIM:101600). The *FGFR2* gene was listed as the top candidate by the rank score. Pfeifer syndrome is an autosomal dominant Mendelian disease and so we applied the autosomal rare dominant model, which further reduced the number of variants to 541 variants, with *FGFR2* still remaining as the top candidate gene.

### Analysis of synthetic whole-genome sequencing dataset on Miller syndrome (MIM: 263750)

We supplemented two known variants (p.G202A and p.G152R) into the *DHODH* gene causing Miller syndrome (MIM: 263750) in the normal exome and applied the rare recessive autosomal disease model filter. The large number of input variants was drastically reduced to 59 variants (28 candidate genes), including the causal gene *DHODH* ranked as the top candidate gene.

### Analysis of targeted resequencing Tourette Syndrome candidate genes

We applied our algorithm to the first study applying next generation sequencing technology in search for genetic susceptibility variants in candidate Tourette Syndrome genes using a set of 382 TS individuals. In this study [33], we identified 17 nonsynonymous variants and experimentally validated five deleterious rare variants. Interestingly, the five variants identified were within the top 6 ranks of our *Variant Ranker* result.

### Family-exome Alzheimer analysis

Our algorithm was applied to describe the genetic findings of two siblings with Alzheimer-type dementia [34]. The exomes of the two siblings were filtered against their unaffected aunt and the variants were ranked using our *Variant Ranker* algorithm. By integrating our ranked results along with other prioritization methods, we were able to get a ranked list of genes which were used for pathway/disease network exploration using our *Result Explorer* module. Our results indicate a set of genes working together in different pathways contributing to the etiology of the complex phenotype.

### Comparison with other web tools

We compare *Variant Ranker* with four similar web-tools using three of our validation datasets, as shown in Table 1. Compared to the other tools, *Variant Ranker* correctly identifies the candidate gene for the respective disorders in all three validation datasets. Feature comparison of the different tools is shown in Table 2. Our tool,*Variant Ranker*, benefits from the simplicity of the ranking formula, which does not necessitate any prior knowledge for the disorder, e.g., knowledge of the inheritance model or required phenotypic/HPO(Human Phenotype Ontology) terms. With default parameters and no model application or special filtering, our tool consistently ranks the candidate genes among the top ten hits that it returns. This is a reasonable cutoff for downstream experimental validation. Web tools like eXtasy [10] that require HPO/phenotypic terms are not competitive with our tool in the case of diagnostic analysis of disorders where no such prior knowledge exists. Unlike *Variant Ranker*, eXtasy [10] is also limited to ranking of non-synonymous variants alone. We also note that wANNOVAR [32] prioritises variants through efficient filtering strategies, but does not produce a ranked list of variants. PhenIX [35] produces a ranked list of genes by calculating clinical similarity using the semantic similarity of HPO terms that areprovided by the user, thus limiting itself to known disease genes.

**Table 1:** Candidate rank comparison using similar web-tools with three of our validation data sets

	Anaemia (*PKLR*,	Pfeifer *(FGFR2)*,	Miller (*DHODH*,
	recessive model)	dominant model)	recessive model)
VariantRanker	1	1	1
eXtasy	436	628	1588
wANNOVAR	12	90	12
PhenIX	1	1	6
wKGGSeq	1	6	3

Candidate gene and inheritance model for respective validation dataset is shown in brackets

**Table 2:** Feature comparison with similar web-tools

		VariantRanker	eXtasy	wANNOVAR	PhenIX	wKGGSeq
Features	Input VCF files	x	x	x	x	x
	Input list of variants	x	-	x	-	x
	Pedigree input	-	-	-	-	x
	Phenotype terms (HPO/OMIM)	-	x	x	x	x
	Result download	x	x	x	-	-
	Excel import	x	x	x	-	-
	Genome browser visualisation	x	-	-	x	x
	Result web storage (shareable links)	x	-	x	-	x
Annotations	Gene information	x	x	x	x	x
	Population frequency	x	-	x	x	x
	Deleteriousness prediction	x	x	x	x	x
	Conservation scores	x	x	x	-	x
	Clinical associations	x	-	x	x	x
Analysis	Variant prioritisation	x	x	x	x	x
	Variant ranking	x	x	-	x	x
	Coding variants	x	x	x	x	x
	Non-coding variants	x	-	x	x	x
	Gene Ranking	x	-	x	x	-
	Functional enrichment analysis	x	-	-	-	-
	Graphical representation	x	-	x	-	x
Filtering	Genotype filtering (Case Control)	x	-	-	-	x
	Variant attributes filtering	x	-	x	-	x
	Inheritance model	x	-	x	x	x
	Mutation count/gene	x	-	-	-	x

## Discussion

### *Variant Ranker*

Input fields include the user’s e-mail address, sample identifier and weighted input parameters between 0 and 1. A default set of weights is provided although the user can change the weights in the input text field (Fig. 3 a) and also deselect databases/algorithms that need to be excluded from the ranking algorithm using the appropriate checkboxes. Users can input a list of variants to prioritize in the form of the *de facto* VCF format or a simple text-based ANNOVAR input format (1-based coordinate system is used with the hg19 human reference build). Our algorithm focuses on biallelic variants and the input file size is restricted to 500 MB. Identified INDELs are excluded from our ranking algorithm although are annotated and provided separately for examination by the user. The output page provides a table of top ranked variants listing 1000 variants at a time and sorted by rank score. We provide a graphical representation for the distribution of the number of SNPs in each chromosome. Below this are a summary of variant counts based on their location and a combined table depicting the summary of scores from CADD, our ranking method and mutation counts per gene (excluding SNPs in non-genic regions i.e. intergenic, upstream or downstream). Users can query the tables on the webpage, sort the output using each of the available columns and also download complete results and import it into Excel. We also provide external links to UCSC genome browser, genecards and ensembl, in order to to provide the user with additional annotation information like gene expression in different tissues through UCSC or additional pathway/disease information from genecards. Results can also be easily shared via URL. The server process fairly quickly under light load. For example 28,000-150,000 variants required about 20-30 minutes to process and a larger file of ~1,000,000 variants took approximately 5 hours to process.

### Prioritization of variants by filtering (*Result Explorer*)

The user can explore the entire ranked volume of data and apply various filtering procedures using the *Result Explorer* module. Options to apply different models of inheritance and also build custom pipelines to filter data using basic SQL queries are available through the advanced query option. The users can search for functional variants and filter by MAF (minor allele frequency) and number of rare mutations per gene. Sample pipelines are provided in the tutorial to filter for (i) variants present in databases like clinVar or GWAS Catalog (ii) functionally important novel variants like exonic (nonsynonymous, stop-loss and stop-gain variants) and splicing sites, (iii) filtering for rare/common variants (MAF filtering) using 1000 genomes, ESP600, and ExAC databases.

### Disease model filtering

For our model filtering criteria, the autosomal dominant filter keeps genes that carry at least one functionally important variant i.e., nonsynonymous, splicing or stopgain/stoploss variant. The autosomal recessive filter keeps genes that carry two or more functionally important variants. The X-recessive filter requires a functionally important variant to be present on the chromosome X positioned gene.

### Case control genotype filtering

For users who want to analyse variants in Case versus Control groups, the *CaseControl filtering* module can be used to filter for case-control genotype differences in order to get a list of variants which can be further ranked using *Variant Ranker*. This module makes use of SnpSift tool [7] to calculate the number of homozygous, heterozygous and total alleles in both Cases and Controls to enable case-control filtering. In this module, processing time for ~1,000,000 variants took only 4 minutes.

### Visualizing functionally enriched terms (*Network Analyser*)

The network web based tool uses RDAVIDWebService package [36] in R to query ontologies. The network is generated using the Cytoscape simple interaction file (SIF) format and is clustered based on Cytoscape’s default web visual style. Gene information is ascribed to hits from the NCBI database. Users can submit top candidate gene symbols (HGNC symbols) and identify overlapping genes from different functionally enriched annotation categories like pathways/ontologies/diseases. Different levels of annotation categories can be explored by filtering using count of genes per category and DAVID *p*-value. The *SNPtoGene* module can be used to map a list of chromosome locations to HGNC gene names.

## Conclusions

We present *Variant Ranker*; a new web server for performing annotation, filtering and ranking of identified genomic variants based on various available databases of genetic variants and facilitating a system for a-priori weight input by the user to identify the most important variants under study. It is a simple and user-friendly web-tool with the ability to rank both coding and non-coding variants by encoding and integrating information from multiple sources. Our tool is intended to help researchers without much computational skills to perform their genomic data analysis.

In contrast to existing methods for prioritization, the present algorithm facilitates the integration of currently available algorithms for prediction and conservation, population frequency, regulatory elements and disease information for each variant based on the user selection. Users can apply case control genotype filtering using the *CaseControl filtering* module. Various filtering strategies for ranked results can be easily applied through the *Result Explorer* module which also facilitates the application of different models of inheritance. Overall, our results indicate that *Variant Ranker* exhibits excellent performance by correctly identifying and ranking the candidate genes for various disorders, as shown with real and synthetic data. Furthermore, using the *Network Analyser* module, users can conduct downstream functional enrichment analysis on top candidate genes and disentangle complex biological associations via network visualization. Our *Variant Ranker* can be applied to various types of sequencing studies, like whole genome or exome studies for both Mendelian and complex disorders. GWAS case-control association and summary statistics data can also be altered to use our tool. We have also applied our algorithm to targeted resequencing data [33] as well as family exome data [34] thus establishing the scope of integrating our methodology with several genomic studies using different experimental designs.

## Availability and requirements

*Variant Ranker* is available at http://paschou-lab.mbg.duth.gr/Software.html. It requires no special or additional data sources, other than the input data from the user. The datasets generated and analysed during the current study are available at http://paschou-lab.mbg.duth.gr/html5up/Examples.html

**Operating system(s):** Platform independent

**Programming language(s):** R, PHP, JavaScript, CSS and HTML

**Other requirements:** Web-browser capable to execute JavaScript/HTML5. Best graphic results on Google Chrome/Mozilla Firefox.

**Any restrictions to use by non-academics:** Contact authors

**Tutorial and Example data:** Available online

## Abbreviations

ChIP-seq: chromatin immunoprecipitation followed by sequencing
HGNC: HUGO Gene Nomenclature Committee
RNA-seq: RNA isolation followed by sequencing
VCF: variant calling format
